# Heterogeneity in COVID-19 vaccine uptake within low-income minority communities: evidence from the watts neighborhood health study

**DOI:** 10.1186/s12889-024-17968-2

**Published:** 2024-02-16

**Authors:** Elizabeth Wong, Ying Liu, Victoria Shier, Ashlesha Datar

**Affiliations:** 1 Center for Economic and Social Research, University of Southern California, 635 Downey Way, VPD, Los Angeles, CA 90089 USA; 2 Sol Price School of Public Policy, Schaeffer Center for Health Policy and Economics, University of Southern California, 635 Downey Way, VPD, Los Angeles, CA 90089 USA

**Keywords:** COVID-19, Vaccination, Low-income, Vaccine attitudes

## Abstract

**Background:**

The literature on disparities in COVID-19 vaccine uptake focuses primarily on the differences between White versus non-White individuals or differences by socioeconomic status. Much less is known about disparities in vaccine uptake *within* low-income, minority communities and its correlates.

**Methods:**

This study investigates disparities in COVID-19 vaccination uptake *within* racial and ethnic minoritized communities with similar socioeconomic backgrounds and built environments, specifically focusing on Black-Hispanic disparities and disparities within the Hispanic community by country of origin. Data are analyzed from the fourth wave (June 2021- May 2022) of the Watts Neighborhood Health Study, a cohort study of public housing residents in south Los Angeles, CA. Linear probability models estimated the association between vaccine uptake and participants’ race/ethnicity, sequentially adding controls for sociodemographic characteristics, health care access and insurance, prior infection, and attitudes towards COVID-19 vaccines. Differences in reasons for vaccination status by race/ethnicity were also tested.

**Results:**

Mexican Hispanic and non-Mexican Hispanic participants were 31% points (95% CI: 0.21, 0.41, *p* < 0.001) and 44% points (95% CI: 0.32, 0.56, *p* < 0.001) more likely to be vaccinated than non-Hispanic Black participants, respectively. The disparity between Black and Hispanic participants was reduced by about 40% after controlling for attitudes towards COVID-19 vaccines. Among Hispanic participants, non-Mexican participants were 13% points (95% CI: 0.03, 0.24, *p* = 0.01) more likely to be vaccinated than Mexican participants, however, these differences were no longer significant after controlling for individual and household characteristics (β = 0.04, 95% CI: -0.07, 0.15, *p* = 0.44).

**Conclusion:**

There are sizeable racial and ethnic COVID-19 vaccination disparities even *within* low-income and minoritized communities. Accounting for this heterogeneity and its correlates can be critically important for public health efforts to ensure vaccine equity.

**Supplementary Information:**

The online version contains supplementary material available at 10.1186/s12889-024-17968-2.

## Introduction

In the United States (U.S.), the COVID-19 pandemic disproportionately affected racial and ethnic minoritized communities [1, 2]. Due in part to socioeconomic factors (e.g., poverty, housing, employment, access to health care), Black and Hispanic individuals had a higher risk of infection, hospitalization, and death relative to White individuals [3, 4]. In April 2021, COVID-19 vaccines became available to all adults in the U.S [5], and the federal government and many state governments made commitments to prioritizing vaccine equity [6]. However, nationwide vaccination efforts have been constrained by inequitable distribution of vaccines and racial, ethnic, and socioeconomic disparities in vaccine uptake [7–9].

The literature on COVID-19 vaccine disparities focuses primarily on the differences between White versus Black and/or Hispanic individuals. Studies have consistently found that Black individuals are significantly less likely than White individuals to be vaccinated, despite being at heightened risk for COVID-19 [10–12]. In the following months after vaccines became widely available to the public, an estimated 77.8% of White individuals had received at least one dose of a vaccine compared to 70.8% of Black individuals [13]. In contrast, findings regarding Hispanic-White vaccination disparities are mixed. While one study reported that Hispanic individuals, like Black individuals, are less likely to be vaccinated than Whites [12], another survey reported no significant differences between Hispanic and White respondents [11]. Further, a third study estimated that Hispanic individuals were 36% more likely to be vaccinated than White individuals [10]. The mixed results among Hispanic populations may be, in part, due to the diversity within this group. Napoles et al. (2021) confirm that, among Hispanic individuals, concerns about vaccine safety, effectiveness, and side effects vary by national origin. For example, Hispanic individuals from Mexico or South America more frequently reported favorable vaccine intentions compared to those with Puerto Rican, Cuban/Dominican, Central American, or other Hispanic ancestry [14].

To further understand and explain differences in vaccination status across racial and ethnic groups, a growing literature is exploring the underlying factors that predict vaccine uptake. Recent studies find that Black and Hispanic adults have higher rates of vaccine hesitancy compared to other racial and ethnic groups [15, 16], and vaccine concerns, particularly related to safety and potential side effects, may partly explain these disparities [17]. Socioeconomic characteristics, such as education and income, are also significant predictors of vaccination status [10, 18]; some studies indicate that Black-White and Hispanic-White disparities in vaccination status can be partly explained by socioeconomic disadvantages among Black and Hispanic communities [12, 19].

But very little attention has been paid to heterogeneity in vaccine uptake *within* marginalized populations. In particular, few studies explore the intersection of race, ethnicity, and socioeconomic status to examine differences in vaccine uptake within and between racial and ethnic communities with similar socioeconomic disadvantages. Since vaccination uptake has plateaued for all racial-ethnic groups since around May 2022 [20], there is a critical need for identifying strategies to make further progress in vaccine uptake. Understanding the heterogeneity within minoritized communities and the factors that contribute to it can offer important insights for designing and targeting interventions for increasing vaccine uptake.

The present study contributes to the literature by examining the heterogeneity in vaccine attitudes and uptake *within* low-income communities, specifically focusing on Black-Hispanic disparities and disparities *within* the Hispanic community by country of origin. Using data collected from residents in urban public housing developments, the study seeks to address three aims: (1) determine whether racial and ethnic disparities in vaccine uptake exist *within* this community, (2) identify factors that explain heterogeneity across racial and ethnic groups, despite their similar socioeconomic backgrounds and exposure to similar geography-based environments, and (3) explore facilitators and barriers to vaccine uptake by race and ethnicity. The findings from this analysis can aid public health practitioners in developing more tailored strategies to improve vaccine uptake in low-income and marginalized communities.

## Methods

### Data and sample

Data are analyzed from the Watts Neighborhood Health Study (WNHS) [21], a cohort study designed to evaluate the impact of a public housing redevelopment in Watts, Los Angeles, California. Between May 2018 and December 2019, WNHS recruited 609 adult and 466 child residents from three public housing sites—Jordan Downs, Nickerson Gardens, and Imperial Courts—and followed them annually over three subsequent waves.

The current study uses survey data from the fourth wave, collected between June 2021 and May 2022, in which adult participants aged 18 and older were asked about their COVID-19 vaccination status, barriers and facilitators to getting a COVID-19 vaccine, attitudes towards COVID-19 vaccines, and pandemic-related hardships. A total of 726 adults participated in this wave, of which 664 adults (76.5%) were originally recruited at baseline, and 62 were child participants at baseline who transitioned to adulthood between baseline and the fourth wave. Of these, 73 adults moved out of the three public housing sites before the fourth wave and were therefore excluded from the analysis. Another 9 participants did not provide information on their vaccination status, the primary outcome of the current study, and one other participant reported race/ethnicity as neither Hispanic nor Black; all 10 of these participants were also excluded from the analysis. Additionally, a total of 27 participants had missing data for some of the covariates described below, and were handled via listwise deletion, resulting in a primary analytic sample of 616 (84.8%) participants.

In the third wave, fielded between June 2020 and April 2021, we asked participants about their access to health care. In the fourth wave analytic sample described above (*n* = 616), a total of 66 (10.7%) did not participate in wave three, reducing the sample to 550 participants. Below, we present the results using the larger analytic sample, however, repeating the same analyses using the smaller, restricted sample with data from both the third and fourth waves shows very similar results (Table S1).

The study was approved by Institutional Review Board at the University of Southern California.

### Measures

#### Vaccination status

The primary outcome of interest was collected by asking participants: “Have you gotten vaccinated for the coronavirus?” (yes/no).

#### Race, ethnicity, and hispanic ancestry

The key predictor of interest was created based on two questions. First, throughout the four waves of data collection we asked participants to identify their race/ethnicity (Hispanic; Black or African American; White; American Indian or Alaska Native; Asian; or Native Hawaiian or other Pacific Islander), allowing participants to select multiple racial or ethnic groups. Second, during the fourth wave, we asked self-identified Hispanic participants about their Hispanic or Latino ancestry or origin using the following categories: Mexican, Mexican American, Chicano; Salvadoran; Guatemalan; Costa Rican; Honduran; Nicaraguan; Panamanian; Puerto Rican; Cuban; Spanish-American (from Spain); Other Hispanic, Latino or Spanish Origin (e.g., Salvadoran, Dominican, Columbian, Guatemalan, Spaniard, Ecuadorian). Because 75.2% of all Hispanic adult participants reported having Mexican ancestry, alone or combined with other origins, we compiled the responses from the two questions and created three race/ethnicity groups: (1) non-Hispanic Black, (2) Hispanic with Mexican ancestry, and (3) Hispanic with non-Mexican ancestry.

#### Attitudes about the COVID-19 vaccines

Six questions were administered to assess attitudes about COVID-19 vaccines. The first four questions asked participants how much they agreed or disagreed with statements that COVID-19 vaccines: (1) Have known harmful side effects; (2) Provide important benefits to society; (3) May lead to illness and death; (4) Are useful and effective. The response options for these first four questions included strongly disagree, disagree, neither agree nor disagree, agree, strongly agree. The remaining two questions asked how much the participants trusted: (5) The process in general (not just for COVID-19) to develop safe vaccines for the public; and (6) The governmental approval process to ensure the COVID-19 vaccine is safe for the public. Response options for these two questions included fully trust, mostly trust, somewhat trust, and do not trust. For each of the six statements, responses were recoded into a binary variable measuring pro-vaccine attitudes (1) versus neutral or anti-vaccine attitudes (0). For example, if an individual “disagreed” or “strongly disagreed” with the statement that the COVID-19 vaccines “have known harmful side effects,” they were coded as having pro-vaccine attitudes. Similarly, if an individual “agreed” or “strongly agreed” with the statement that COVID-19 vaccines “provide important benefits to society,” they were considered as having pro-vaccine attitudes.

Due to collinearity between some questions measuring COVID-19 attitudes, our primary regression analyses include three of these six variables that measure three distinct domains: perceived benefit (COVID-19 vaccine provides important benefits to society); perceived harm (COVID-19 vaccine may lead to illness and death); and perceived trust (trust the process in general (not just for COVID-19) to develop safe vaccines for the public). Sensitivity analyses show similar results when these three variables are replaced with the other three variables in the regression analysis (Table S2).

#### Prior COVID-19 infection

Information on participants’ COVID-19 infection status was collected by asking: “Do you think you’ve been infected with the coronavirus?” (yes/no).

#### COVID-19 related hardships

Participants were also asked to rate “how much of a problem were each of the following things during the past month,” on a scale of 1 to 10, where 1 means not a problem at all and 10 means a big problem: *maintaining your income; staying connected with family; staying connected with friends; grocery shopping and access to food; having enough food for you and your family; going out to eat or for entertainment; and being able to exercise and physically active*. Exploratory factor analysis showed a strong single factor structure among these items, with a single strong eigenvalue of 3.20 and a Cronbach’s alpha of 0.85. We used an average score in this analysis as it was almost perfectly correlated with the factor score (*r* = 0.99).

#### Health conditions and functional limitations

Because participants’ experiences during the pandemic and attitudes about COVID-19 vaccines may depend on their health status, we included three indicators in our analysis to measure health conditions and functional limitations: (1) whether the participant was ever diagnosed with any cardiometabolic conditions (heart disease, diabetes type 2, high blood sugar, hypertension, high cholesterol, or obesity; (2) whether he/she had “serious difficulty walking, climbing stairs, bathing, dressing or doing errands alone” (never, some of the time, a moderate amount of time, and most of the time, with the latter three options grouped to create an “any limitation” indicator); and (3) whether he/she had obesity. The obesity status was defined as having body mass index greater than or equal to 30.0. Trained staff collected weight and height measurements using a Tanita UM-081 digital scale and Charder HM200P Portstad Portable Stadiometer, respectively. A small portion of the participants (< 5%) did not have measurement data in the fourth wave, and therefore their weight and height information from earlier waves were used in the analysis.

#### Access to health insurance and health care

Participants’ attitudes about the COVID-19 vaccines might also reflect, to some degree, their general experience with the medical system. As such we included in the analysis participants’ self-reports on their access to health care during the earlier stage of the pandemic. Specifically, we asked: (1) “Do you currently have any health insurance coverage or some way to pay for your health care?” (yes/no); and (2) “Is there a place that you usually go to when you are sick or need advice about your health?” (yes/no). Participants were also asked where they received such care, including major hospitals and health centers nearby, as well as private practices. These responses were used to derive an indicator for whether a participant had access to medical professionals when needed. These questions were only asked in the prior year’s survey.

#### Time of the primary survey

Data was collected from participants over a year long period, during which the COVID-19 infection rate and the vaccination policy in Los Angeles County were rapidly changing. To address this we split the data collection window into two periods: (1) June to November 2021, soon after the COVID-19 vaccines became available to all adults in Los Angeles County in April 2021 and when the infection rate was relatively low; and (2) December 2021 to June 2022, during and after the Omicron surge when the infection rate peaked and went down [22]. We chose this breakdown because people’s attitudes about COVID-19 vaccines might have varied depending on the disease environment. Throughout the entire data collection period, participation was fairly balanced across race/ethnicity groups (Figure S1).

#### Demographic and household characteristics

Demographic information of the participants was also collected, including age (18–34, 35–54, 55 and older), gender (male/female), education (less than high school, high school, more than high school), whether married or living as married (yes/no), and in which country the participant was born (U.S./outside U.S.). Additional household information included income (less than $10,000, $10,000-$19,999, $20,000 and more), whether any household member worked for pay (yes/no), and the number of children in the household (none, one or two, three or more).

#### Reasons for vaccination status

Participants who were vaccinated were asked, “What is the main reason you chose to get vaccinated?”. Responses were grouped into four categories: (1) work or school mandate; (2) to protect oneself; (3) to protect family or others; and (4) other/unknown. Participants who were not vaccinated were asked, “What is your biggest barrier to getting the coronavirus vaccine?”. Responses were grouped into six categories: (1) distrust in COVID-19 or the vaccine; (2) concerns about vaccine safety; (3) underlying health condition(s); (4) waiting for additional information or unsure; (5) no barriers; and (6) other/unknown.

### Analysis

To estimate the association between the racial and ethnic groups and vaccination status, we used a “build” regression approach that adds blocks of covariates, one at a time, to predict vaccination status. We estimate linear probability models for easy interpretability of coefficients; logistic models yielded similar results (Table S3). Starting with a model with race/ethnicity groups and survey time period covariates, we added: (1) demographic and household characteristics, (2) prior infection with COVID-19, (3) health conditions and functional limitations, (4) COVID-19 related hardships in the past month, (5) attitudes about COVID-19 vaccines, and (6) access to health insurance and health care. Although the majority (56%) of participants came from single-respondent households, standard errors were clustered at the household level to account for household-level effects in cases where multiple respondents resided in the same household. Chi-squared tests were used to compare reasons for vaccination status between non-Hispanic Black and both Hispanic groups combined (due to sample size concerns). All analyses were conducted using Stata 16.1.

## Results

Table 1 presents the descriptive statistics for the analysis sample, overall and by race/ethnicity. Overall, about 22% of the sample was 55 years or older and 75% was female. Nearly one-third of the sample (27.9%) had less than a high school degree, 38.2% had a household income of less than $10,000 per year, and 40% were born outside the U.S. In addition, most of the sample had health insurance (86.4%) and access to a medical professional (88.8%).

**Table 1:** Sample characteristics, overall and by race, ethnicity, and hispanic ancestry

		Overall (*n*=616)	Non-Hispanic Black (*n*=174)	Hispanic, Mexican Ancestry (*n*=343)	Hispanic, Non-Mexican Ancestry (*n*=99)	***p***-value
		n (%) or mean (sd)				
**Demographic and Household Characteristics**						
Age						
	18-34	238 (38.6)	49 (28.2)	163 (47.5)	26 (26.3)	<0.001
	35-54	242 (39.3)	75 (43.1)	129 (37.6)	38 (38.4)	
	55+	136 (22.1)	50 (28.7)	51 (14.9)	35 (35.4)	
Sex						
	Male	154 (25.0)	36 (20.7)	93 (27.1)	25 (25.3)	0.28
	Female	462 (75.0)	138 (79.3)	250 (72.9)	74 (74.8)	
Education						
	Less than high school	172 (27.9)	19 (10.9)	110 (32.1)	43 (43.4)	<0.001
	High school	250 (40.6)	90 (51.7)	135 (39.4)	25 (25.3)	
	More than high school	194 (31.5)	65 (37.4)	98 (28.6)	31 (31.3)	
Household income						
	Less than $10,000	235 (38.2)	93 (53.5)	108 (31.5)	34 (34.3)	<0.001
	$10,000-$19,999	159 (25.8)	42 (24.1)	95 (27.7)	22 (22.2)	
	$20,000 or greater	222 (36.0)	39 (22.4)	140 (40.8)	43 (43.4)	
Survey completion date						
	June - November 2021	483 (78.4)	134 (77.0)	271 (79.0)	78 (78.8)	0.868
	December 2021 or later	133 (21.6)	40 (23.0)	72 (21.0)	21 (21.2)	
Any household member works for pay						
	No	153 (24.8)	82 (47.1)	44 (12.8)	27 (27.3)	<0.001
	Yes	463 (75.2)	92 (52.9)	299 (87.2)	72 (72.7)	
Married or living as married						
	No	473 (76.8)	157 (90.2)	251 (73.2)	65 (65.7)	<0.001
	Yes	143 (23.2)	17 (9.8)	92 (26.8)	34 (34.3)	
Number of children in the household						
	None	210 (34.1)	88 (50.6)	85 (24.8)	37 (37.4)	<0.001
	One or two	268 (43.5)	62 (35.6)	169 (49.3)	37 (37.4)	
	Three or more	138 (22.4)	24 (13.8)	89 (26.0)	25 (25.3)	
Country of birth						
	U.S. born	367 (59.6)	173 (99.4)	168 (49.0)	26 (26.3)	<0.001
	Foreign born	249 (40.4)	1 (0.6)	175 (51.0)	73 (73.7)	
**Ever infected with COVID-19**						
	No	426 (69.2)	151 (86.8)	216 (63.0)	59 (59.6)	<0.001
	Yes	190 (30.8)	23 (13.2)	127 (37.0)	40 (40.4)	
**Health conditions and functional limitations**						
Ever diagnosed with cardiometabolic condition						
	No	296 (48.1)	82 (47.1)	177 (51.6)	37 (37.4)	0.043
	Yes	320 (52.0)	92 (52.9)	166 (48.4)	62 (62.6)	
Have obesity						
	No	255 (41.4)	79 (45.4)	141 (41.1)	35 (35.4)	0.265
	Yes	361 (58.6)	95 (54.6)	202 (58.9)	64 (64.7)	
Difficulties with activities of daily living						
	No	491 (79.7)	132 (75.9)	287 (83.7)	72 (72.7)	0.019
	Yes	125 (20.3)	42 (24.1)	56 (16.3)	27 (27.3)	
**COVID-19 related experiences**		4.99 (2.35)	4.63 (2.48)	5.03 (2.34)	5.47 (2.05)	0.016
**Perceptions about COVID-19 vaccines**						
Trust the process to develop a safe COVID-19 vaccine						
	Neutral or anti-vaccine	356 (57.8)	135 (77.6)	177 (51.6)	44 (44.4)	<0.001
	Pro-vaccine	260 (42.2)	39 (22.4)	166 (48.4)	55 (55.6)	
COVID-19 vaccine provides important benefits to society						
	Neutral or anti-vaccine	261 (42.4)	106 (60.9)	128 (37.3)	27 (27.3)	<0.001
	Pro-vaccine	355 (57.6)	68 (39.1)	215 (62.7)	72 (72.7)	
COVID-19 vaccine may lead to illness and death						
	Neutral or anti-vaccine	388 (63.0)	129 (74.1)	203 (59.2)	56 (56.6)	0.001
	Pro-vaccine	228 (37.0)	45 (25.9)	140 (40.8)	43 (43.4)	
**Access to health insurance and health care**						
Health insurance						
	No	75 (13.6)	13 (8.3)	47 (15.5)	15 (16.5)	0.072
	Yes	475 (86.4)	143 (91.7)	256 (84.5)	76 (83.5)	
Health care						
	No	62 (11.3)	9 (5.7)	43 (14.2)	10 (11.0)	0.025
	Yes	489 (88.8)	148 (94.3)	260 (85.8)	81 (89.0)	
**COVID-19 vaccination status**						
	Not vaccinated	241 (39.1)	110 (63.2)	112 (32.7)	19 (19.2)	<0.001
	Vaccinated	375 (68.9)	64 (36.8)	231 (67.4)	80 (80.8)	

With respect to racial/ethnic composition, 28.2% were non-Hispanic Black, 55.7% were Hispanic with Mexican ancestry, and 16.1% were Hispanic with non-Mexican ancestry. There were several significant differences in demographic and household characteristics by race/ethnicity. For example, education and household income varied significantly by race/ethnicity groups (*p* < 0.001 for both). Fewer non-Hispanic Black participants (10.9%) had less than high school education than Hispanic participants (32.1% of Mexican and 43.4% of non-Mexican Hispanic participants). More non-Hispanic Black participants had a household income of less than $10,000 than Hispanic participants (53.5% of non-Hispanic Black, 31.5% of Mexican Hispanic, 34.3% of non-Mexican Hispanic participants). Non-Hispanic Black participants were also less likely to have a household member who works for pay (52.9% vs. 87.2% and 72.7% for Mexican Hispanic and non-Mexican Hispanic participants, respectively; *p* < 0.001) or be foreign born than other participants (0.6% vs. 51.0% and 73.7% for Mexican Hispanic and non-Mexican Hispanic participants, respectively; *p* < 0.001).

Vaccination status and vaccine attitudes were significantly different between non-Hispanic Black participants and the Hispanic participants; 36.8% of non-Hispanic Black participants were vaccinated, compared to 67.4% of Mexican Hispanic and 80.8% for non-Mexican Hispanic participants (*p* < 0.001). Hispanic participants were also more likely to have pro-vaccine attitudes. About half of Mexican (51.6%) and non-Mexican (44.4%) Hispanic participants mostly trusted or somewhat trusted the process to develop a safe vaccine, compared to less than one-fourth of non-Hispanic Black participants (22.4%) (*p* < 0.001). There were similar patterns for attitudes about vaccines providing an important benefit to society and about vaccines not leading to illness or death.

Table 2 presents estimates from linear probability models for vaccination status, adding one block of covariates at a time. As shown in Model 0, before controlling for any covariates other than timing of the survey, vaccination status varied significantly by race/ethnicity. Mexican Hispanic and non-Mexican Hispanic participants were 31% points (*p* < 0.001) and 44% points (*p* < 0.001) more likely to be vaccinated than non-Hispanic Black participants. The difference in vaccination among Hispanic participants (shown at the bottom of the table) was also relatively large; non-Mexican Hispanic participants were 13% points (*p* = 0.011) more likely to be vaccinated than Mexican participants. After adding demographic and household characteristics (Model 1), the differences in vaccination status between non-Hispanic Black participants and Hispanic participants persisted. Hispanic participants (both Mexican and non-Mexican Hispanic) were at least 30% points more likely to be vaccinated than non-Hispanic Black participants. However, the difference in vaccination status between Hispanic participants based on ancestry was reduced; non-Mexican Hispanic participants were only 4% points more likely to be vaccinated than Mexican Hispanic participants (*p* = 0.417). Beyond race/ethnicity, the only demographic or household characteristic that was significantly associated with vaccination status was age; participants 55 years or older were 34% points more likely to be vaccinated than participants 18–34 years old (*p* < 0.001).

**Table 2:** Survey completed row label should not be indented with the race/ethnicity categories

		Model 0	Model 1	Model 2	Model 3	Model 4	Model 5	Model 6
Race, Ethnicity, Ancestry (ref. = Non-Hispanic Black)								
	Hispanic, Mexican ancestry	0.31*** (0.21,0.41)	0.30*** (0.18,0.42)	0.30*** (0.18,0.42)	0.31*** (0.19,0.42)	0.31*** (0.19,0.43)	0.19*** (0.09,0.29)	0.23*** (0.12,0.33)
	Hispanic, non-Mexican ancestry	0.44*** (0.32,0.56)	0.35*** (0.20,0.49)	0.35*** (0.20,0.49)	0.35*** (0.20,0.49)	0.35*** (0.20,0.50)	0.22*** (0.09,0.34)	0.22** (0.09,0.36)
	Survey completed December 2021 or later	0.09 (-0.00,0.19)	0.08 (-0.01,0.17)	0.08 (-0.01,0.17)	0.08 (-0.01,0.18)	0.08 (-0.01,0.18)	0.08 (-0.01,0.16)	0.08 (-0.00,0.17)
Age (ref. = 18-34)								
	35-54		0.10 (-0.01,0.20)	0.10 (-0.01,0.20)	0.10 (-0.01,0.20)	0.10 (-0.01,0.20)	0.05 (-0.03,0.14)	0.06 (-0.03,0.15)
	55+		0.34*** (0.21,0.47)	0.34*** (0.21,0.47)	0.34*** (0.19,0.48)	0.33*** (0.19,0.48)	0.26*** (0.13,0.38)	0.24*** (0.11,0.37)
Female			0.01 (-0.08,0.10)	0.01 (-0.08,0.10)	0.01 (-0.08,0.10)	0.01 (-0.08,0.10)	0.07 (-0.01,0.15)	0.04 (-0.04,0.12)
Education (ref. = Less than high school)								
	High school		0.00 (-0.10,0.09)	0.00 (-0.10,0.10)	0.00 (-0.10,0.09)	0.00 (-0.10,0.09)	0.00 (-0.07,0.08)	-0.01 (-0.09,0.08)
	More than HS		0.00 (-0.11,0.11)	0.00 (-0.11,0.11)	-0.01 (-0.12,0.10)	0.00 (-0.11,0.11)	0.04 (-0.05,0.12)	0.04 (-0.06,0.13)
Household Income (ref. = Less than $10,000)								
	$10,000-$19,999		0.03 (-0.08,0.14)	0.03 (-0.08,0.14)	0.03 (-0.08,0.14)	0.03 (-0.08,0.14)	-0.01 (-0.10,0.08)	-0.02 (-0.11,0.07)
	$20,000 or greater		0.06 (-0.05,0.17)	0.06 (-0.05,0.17)	0.06 (-0.05,0.17)	0.06 (-0.05,0.17)	0.02 (-0.06,0.11)	0.05 (-0.04,0.14)
Any household member works for pay			-0.01 (-0.12,0.09)	-0.01 (-0.12,0.09)	-0.02 (-0.13,0.09)	-0.02 (-0.13,0.09)	-0.01 (-0.10,0.07)	-0.03 (-0.11,0.06)
Married or living as married			0.06 (-0.04,0.16)	0.06 (-0.04,0.16)	0.06 (-0.04,0.16)	0.06 (-0.04,0.16)	0.02 (-0.07,0.11)	0.00 (-0.09,0.09)
Number of children in the household (ref. = No children)								
	One or two children in the household		0.02 (-0.09,0.13)	0.02 (-0.09,0.13)	0.02 (-0.09,0.13)	0.02 (-0.09,0.13)	-0.01 (-0.10,0.09)	-0.01 (-0.10,0.09)
	Three or more children in the household		-0.02 (-0.16,0.11)	-0.02 (-0.16,0.11)	-0.03 (-0.17,0.11)	-0.03 (-0.17,0.11)	-0.01 (-0.12,0.10)	-0.01 (-0.12,0.11)
Foreign born			0.08 (-0.05,0.20)	0.08 (-0.05,0.20)	0.08 (-0.05,0.20)	0.08 (-0.05,0.20)	0.02 (-0.08,0.13)	0.04 (-0.07,0.15)
Ever been infected with COVID-19				-0.01 (-0.09,0.08)	0.00 (-0.09,0.08)	0.00 (-0.09,0.08)	0.02 (-0.05,0.10)	0.02 (-0.05,0.10)
Ever been diagnosed with cardiometabolic condition					0.03 (-0.05,0.12)	0.03 (-0.05,0.12)	-0.01 (-0.08,0.06)	-0.03 (-0.11,0.04)
Have obesity					-0.02 (-0.10,0.06)	-0.02 (-0.09,0.06)	0.00 (-0.07,0.07)	0.01 (-0.06,0.08)
Difficulties with activities of daily living					-0.04 (-0.14,0.06)	-0.04 (-0.14,0.06)	-0.05 (-0.13,0.03)	-0.03 (-0.12,0.05)
COVID-19 problem scale						0.00 (-0.02,0.01)	0.00 (-0.01,0.01)	0.00 (-0.02,0.01)
Trust the process to develop safe COVID-19 vaccine							0.19*** (0.11,0.27)	0.18*** (0.10,0.26)
COVID-19 vaccine provides important benefits to society							0.29*** (0.20,0.37)	0.27*** (0.18,0.36)
Disagree that COVID-19 vaccine leads to illness or death							0.17*** (0.10,0.24)	0.15*** (0.08,0.23)
Health insurance								0.08 (-0.03,0.19)
Access to health care								0.09 (-0.03,0.22)
Constant		0.35*** (0.27,0.43)	0.18* (0.01,0.35)	0.18* (0.01,0.36)	0.19* (0.01,0.37)	0.21* (0.02,0.39)	0.01 (-0.15,0.16)	-0.11 (-0.30,0.09)
N		616	616	616	616	616	616	550
R2		0.112	0.209	0.209	0.211	0.211	0.431	0.432
Race, Ethnicity, Ancestry (ref. = Hispanic, Mexican ancestry) ^a^								
	Hispanic, non-Mexican ancestry	0.13* (0.03,0.24)	0.04 (-0.07,0.15)	0.04 (-0.07,0.15)	0.04 (-0.07,0.15)	0.04 (-0.07,0.15)	0.03 (-0.06,0.12)	0.00 (-0.10,0.09)

* *p* < 0.05, ** *p* < 0.01, *** *p* < 0.001

^a^ Estimates comparing Hispanic Mexican and Hispanic non-Mexican individuals are calculated from the same regression models estimated above within the same column, using Mexican Hispanic as the reference group rather than non-Hispanic Black

Adding covariates for whether the participant had a prior infection of COVID-19 (model 2), potential health conditions and functional limitations (model 3), and COVID-19 related hardships (model 4) did not explain the gap in vaccination between non-Hispanic Black and Hispanic participants. The difference in vaccination between non-Mexican Hispanic and Mexican Hispanic participants remained small and non-significant (4% points).

In Model 5, attitudes about the COVID-19 vaccine (trust to develop a safe vaccine, vaccine provides important benefit, and vaccine does not lead to illness or death) were added to the model. After controlling for these attitudes, disparities in vaccination between non-Hispanic Black and Hispanic participants were reduced by about 40%; Mexican and non-Mexican Hispanic participants were 19 and 22% points more likely to be vaccinated compared to non-Hispanic Black participants, respectively. Vaccine attitudes were significantly associated with vaccination. Fully trusting or mostly trusting the process to develop a safe vaccine was associated with 19% points higher likelihood of being vaccinated (*p* < 0.001). Strongly or somewhat agreeing with vaccines providing an important benefit to society was associated with 29% points higher likelihood of being vaccinated (*p* < 0.001), and strongly or somewhat disagreeing with vaccine leading to illness or death was associated with 17% points higher likelihood of being vaccinated (*p* < 0.001). Finally, adding health insurance and health care access variables (model 6), did not further explain the gap in vaccination status between non-Hispanic Black and Hispanic (Mexican or non-Mexican) participants. Predicted vaccination rates for each racial and ethnic group from each model are reported in Table S3.

**Table 3:** Reasons for vaccination status

		Overall	Non-Hispanic Black	Hispanic	Pearson’s chi-square test ***p***-value
Main reason for getting vaccinated (asked to participants who were vaccinated)					*p*=0.09
	Work or school mandate	11.8%	15.6%	11.0%	
	Protect oneself	48.7%	54.7%	47.4%	
	Protect family or others	30.0%	17.2%	32.6%	
	Other/unknown	9.6%	12.5%	9.0%	
N		374	64	310	
Biggest barrier to getting vaccinated (asked to participants who were not vaccinated)					*p*=0.81
	Distrust in COVID-19 or the vaccine	34.6%	36.4%	33.1%	
	Concerns about vaccine safety	26.3%	29.1%	23.9%	
	Underlying health condition(s)	7.5%	7.3%	7.7%	
	Uncertainty (e.g., waiting, need more information)	8.3%	6.4%	10.0%	
	No barriers	10.8%	10.0%	11.5%	
	Other/unknown	12.5%	10.9%	13.9%	
N		240	110	130	

Table 3 presents the reasons for vaccination status for the full sample and by race/ethnicity. Overall, protecting oneself (48.7%) and protecting family or others (30.0%) were the two most cited reasons for vaccination among adults who were vaccinated. Among those who were not vaccinated, 34.6% cited distrust in COVID-19 or the vaccine as the biggest reason for their decision. The second most cited reason was concerns about vaccine safety (26.3%). When comparing racial/ethnic groups, there were some notable differences although they were not statistically significant. Compared to non-Hispanic Black participants, a notably higher percentage of Hispanic participants reported being vaccinated to protect their family or others (17.2% and 32.6%, respectively). A slightly higher percentage of non-Hispanic Black participants reported distrust (36.4%) and concerns about vaccine safety (29.1%) compared to Hispanic participants (33.1% and 23.9%, respectively).

## Discussion

Since the start of the pandemic, research has extensively documented racial, ethnic, and socioeconomic disparities in COVID-19 vaccine attitudes and vaccination status [15, 23, 24]. However, few of these studies have focused on the intersection of race, ethnicity, and socioeconomic status to understand vaccine disparities *within* low-income minoritized communities. The current study seeks to fill this research gap by examining the heterogeneity in vaccine attitudes and uptake within low-income and socially disadvantaged communities, specifically focusing on Black-Hispanic disparities and disparities within the Hispanic community by country of origin.

Our analyses reveal substantial disparities in vaccination status between low-income Black, Mexican Hispanic, and non-Mexican Hispanic groups. We find that low-income Hispanic individuals, regardless of their country of origin, are more likely than low-income Black individuals to receive the COVID-19 vaccine. Specifically, Hispanic individuals with Mexican ancestry are 31% points more likely than Black individuals to be vaccinated. The difference is even greater for non-Mexican Hispanic individuals, who are 44% points more likely thank Black individuals to be vaccinated.

The vaccination gap between low-income Black and Hispanic individuals residing in public housing developments is substantially larger than Black-Hispanic vaccination gaps reported in other studies which use more heterogeneous samples [12, 13]. For example, a study of registered voters in California reported that 65% of Black respondents and 67% of Hispanic respondents reported receiving at least one dose of a COVID-19 vaccine [12]. Using nationally representative data from the Household Pulse Survey, another study also reported minimal differences between the estimated vaccination rates of Hispanic (74%) and Black populations (71%) [13]. It is possible that vaccination disparities between Black and Hispanic communities are concentrated within low-income groups. Therefore, evidence of these differences may be muted in studies that survey more diverse Black and Hispanic samples from a range of economic backgrounds.

Perhaps due to previous research that has demonstrated only slight differences between Black and Hispanic vaccination rates, little attention has been given to understanding vaccination disparities between these two groups. Most research investigating racial and ethnic vaccination disparities compare White individuals with minority groups. These studies commonly suggest that socioeconomic disadvantages (e.g., education, income, location) create structural and geographic barriers to COVID-19 vaccines for minority populations [12, 19, 25]. However, there are two indications to suggest that these barriers do not explain the sizeable Black-Hispanic COVID-19 vaccine disparity we find among low-income communities. First, our sample was recruited from three similar public housing developments in one south Los Angeles neighborhood, and there is little evidence of variation in vaccine accessibility within that community. Around May 2021, after vaccines became widely available to the public, community organizations, churches, and health care providers began partnering to bring mobile clinics and pop-up vaccination sites to the Watts neighborhood. Second, contrary to the literature, our results indicate that individual barriers, such as educational attainment, income, and health insurance coverage, do not significantly predict vaccination status. In fact, we find that Hispanic individuals, despite having lower levels of education, are actually more likely to be vaccinated than Black individuals. Moreover, access to health care (89%) and health insurance (86%) is also generally high in our sample, with relatively less variability across racial-ethnic minority groups, which might also explain why it did not predict vaccination status. Furthermore, recent evidence based on more representative samples from the 2022 US household pulse survey also suggests that insurance status was not predictive of COVID-19 vaccination status [10], perhaps due to its free availability and efforts to rapidly increase access both within and outside of traditional health care systems (e.g., via mass vaccination sites).

Rather, the results from these analyses demonstrate that attitudes about COVID-19 vaccines significantly predict vaccination status and play a large role in explaining the Black-Hispanic vaccine disparity. After accounting for individual attitudes about the COVID-19 vaccine, the Black-Hispanic (Mexican ancestry) disparity was reduced by nearly 40%, and the Black-Hispanic (non-Mexican ancestry) was reduced by 50%. These findings may be driven, in part, by more positive attitudes about the COVID-19 vaccines among Hispanic communities compared to Black communities. Indeed, in our sample, 48% of Hispanic individuals of Mexican ancestry and 56% of Hispanic individuals of non-Mexican ancestry trusted the government to develop a safe COVID-19 vaccine, compared to only 22% of Black individuals. This trend is consistent with other studies from California and the U.S. which show that Black individuals more frequently report negative vaccine attitudes and perceptions relative to Hispanic individuals [26–28].

While our analyses suggest that vaccine attitudes explain a notable portion of the Black-Hispanic vaccination disparity, our models were unable to explain the full difference. Even after accounting for age, sex, education, employment status, household composition, health status, vaccine attitudes, health insurance coverage, and health care access, we find that Hispanic individuals are still roughly 20% points more likely to be vaccinated than Black individuals. This indicates that there are other factors leading to vaccine disparities between Hispanic and Black individuals.

One potential explanation could be industry and occupational differences between Black and Hispanic workers in our sample, and varying exposure to COVID-19 mandates. Using data from the 2019 American Community Survey, a recent paper reported that Hispanic workers are employed in the U.S. health care workforce at slightly higher rates than Black workers (18.2% and 12.1%, respectively) [29]. In California, health care workers were required to be fully vaccinated starting August 2021. If Hispanic participants were employed more frequently in the health care or any other sector that mandated COVID-19 vaccination, they may have been more likely to receive the vaccine. However, our data shows that Black and Hispanic individuals in our sample had similar experiences with workplace vaccine mandates, although Black participants were less likely to have a working household member than Hispanic participants. Among respondents who were vaccinated, we find no significant difference between the number of non-Hispanic Black individuals (16%) and Hispanic individuals (11%) who reported workplace mandates as the primary reason for being vaccinated. This suggests that industry and occupational differences likely do not explain vaccine disparities within our sample.

Other studies have also considered the role that discrimination plays in creating and perpetuating medical mistrust, and the effect that this process has in explaining vaccine hesitancy and uptake [30], particularly among racial and ethnic minoritized communities [12, 15, 31]. Because our study did not collect this information from participants, we were unable to examine how these factors relate to disparities in vaccine attitudes or vaccine uptake between low-income Black and Hispanic individuals. Nonetheless, future studies may consider investigating whether and how experiences with discrimination and medical mistrust explain disparities in vaccination attitudes and behaviors between these two marginalized groups. It is also important for future research to consider how institutional efforts to improve trust and communication between communities and health care professionals could support vaccine equity [32].

A unique contribution of these analyses was the ability to look at vaccine disparities within the Hispanic community. Based on a previous study that documented differences in vaccine intention and concerns between Hispanic individuals depending on national origin [14], we expected to see differences in vaccination status between Hispanic individuals with Mexican ancestry and Hispanic individuals with non-Mexican ancestry. In our base model, we do find evidence to suggest significant differences, where Hispanic individuals with non-Mexican ancestry are 13% points more likely to be vaccinated than Hispanic individuals with Mexican ancestry. This contrasts with findings from Napoles et al. (2021) who found that individuals of Mexican origin more frequently reported positive vaccine intention compared to individuals from other Hispanic origins including those of Puerto Rican, Cuban/Dominican, and Central American descent [14]. Interestingly, after accounting for individual and household characteristics, any differences between non-Mexican and Mexican Hispanic individuals are attenuated and no longer significant.

These results may be driven by differences within the non-Mexican Hispanic sample—because over three quarters of Hispanic participants were of Mexican ancestry, we grouped Hispanics with Salvadoran, Guatemalan, Costa Rican, Honduran, Nicaraguan, Panamanian, Puerto Rican, Cuban, or other ancestry into a single comparison group. The small sample sizes of these other groups limit our ability to conduct a more detailed analyses. However, as we know from the literature, these are all heterogenous Hispanic communities and have different beliefs and experiences related to the pandemic and the COVID-19 vaccines. Future studies with access to a more diverse sample of Hispanic individuals should consider exploring more deeply the differences in vaccination rates based on national origin.

Our study has some limitations that should be considered when interpreting these results. First, our survey broadly asked participants whether they had been vaccinated for the coronavirus but did not specifically ask whether they had received a single dose, the full primary series, or any booster. Participants may have interpreted this question differently. Second, vaccination status was self-reported during a phone-based survey and may be subject to social desirability bias. Third, our sample of low-income public housing residents was recruited from one urban community in south Los Angeles. Given that states and counties took unique approaches to managing the virus and administering vaccines, these results may not be generalizable to low-income public housing residents in other regions, other states, or even other cities within California.

In conclusion, this study advances the current literature on racial and ethnic differences in COVID-19 vaccine attitudes and uptake. Previous studies have largely framed vaccine inequity around differences in vaccine uptake between White communities and Black and/or Hispanic communities, thus the proposed solution is often a blanket call for targeted public health messaging to increase vaccination rates among minority populations, generally. However, this study reveals that there are prominent racial and ethnic disparities in vaccine attitudes and vaccine uptake within minoritized groups. The results highlight the importance of acknowledging heterogeneity within marginalized communities, supporting the idea that there is no “one-size-fits-all” solution to achieving vaccine equity [33]—tailored approaches for Mexican American communities may not be effective in other Hispanic communities. Identifying and understanding these differences are critical to developing more nuanced public health messaging to improve vaccine equity, not just for COVID but also for vaccines more generally.

### Electronic supplementary material

Below is the link to the electronic supplementary material.

Supplementary Material 1

## Data Availability

The de-identified individual-level dataset used during this current study can be made available from the corresponding author upon reasonable request.
