# Supplementary material for: Heterogeneity in COVID-19 vaccine uptake within low-income minority communities: evidence from the watts neighborhood health study
Source: BMC Public Health. 2024 Feb 16;24:503. doi: 10.1186/s12889-024-17968-2 (PMC10873997; doi:10.1186/s12889-024-17968-2)
Supplement: Supplementary file 1 — Supplementary Material 1 [file 12889_2024_17968_MOESM1_ESM.docx]

| **Table S1. Regression Coefficients from Linear Probability Models Predicting Vaccination Status (Using Restricted Analytic Sample of Participants with Wave 3 and Wave 4 Data)** | | | | | | | | |
| --- | --- | --- | --- | --- | --- | --- | --- | --- |
|  |  | Model 0 | Model 1 | Model 2 | Model 3 | Model 4 | Model 5 | Model 6 |
| Race, Ethnicity, Ancestry (ref. = Non-Hispanic Black) | | 0.34*** (0.24,0.44) | 0.33*** (0.21,0.46) | 0.33*** (0.21,0.46) | 0.33*** (0.21,0.46) | 0.33*** (0.21,0.46) | 0.23*** (0.12,0.34) | 0.24*** (0.13,0.35) |
|  | Hispanic, Mexican ancestry | 0.45*** (0.32,0.57) | 0.35*** (0.19,0.50) | 0.35*** (0.19,0.51) | 0.35*** (0.19,0.51) | 0.35*** (0.19,0.51) | 0.22*** (0.09,0.35) | 0.23*** (0.10,0.36) |
|  | Hispanic, non-Mexican ancestry | 0.11* (0.01,0.20) | 0.09 (-0.00,0.19) | 0.09 (-0.00,0.19) | 0.09 (-0.00,0.19) | 0.09 (-0.01,0.19) | 0.11* (0.02,0.20) | 0.10* (0.02,0.19) |
|  | Survey completed December 2021 or later |  |  |  |  |  |  |  |
| Age (ref. = 18-34) | |  |  |  |  |  |  |  |
|  | 35-54 |  | 0.10 (-0.01,0.21) | 0.10 (-0.01,0.21) | 0.10 (-0.01,0.21) | 0.10 (-0.01,0.21) | 0.03 (-0.06,0.12) | 0.03 (-0.07,0.12) |
|  | 55+ |  | 0.33*** (0.20,0.46) | 0.33*** (0.19,0.46) | 0.32*** (0.18,0.47) | 0.32*** (0.18,0.46) | 0.20** (0.08,0.32) | 0.20** (0.07,0.32) |
| Female | |  | 0.01 (-0.08,0.10) | 0.01 (-0.08,0.10) | 0.01 (-0.08,0.10) | 0.01 (-0.08,0.11) | 0.04 (-0.05,0.12) | 0.02 (-0.06,0.10) |
| Education (ref. = Less than high school) | |  |  |  |  |  |  |  |
|  | High school |  | -0.02 (-0.12,0.09) | -0.02 (-0.12,0.09) | -0.02 (-0.12,0.09) | -0.02 (-0.12,0.09) | 0.01 (-0.07,0.09) | 0.01 (-0.07,0.09) |
|  | More than HS |  | 0.00 (-0.11,0.12) | 0.00 (-0.11,0.12) | 0.00 (-0.11,0.12) | 0.00 (-0.11,0.12) | 0.08 (-0.01,0.18) | 0.08 (-0.02,0.17) |
| Household Income (ref. = Less than $10,000) | |  |  |  |  |  |  |  |
|  | $10,000-$19,999 |  | 0.02 (-0.10,0.13) | 0.02 (-0.10,0.13) | 0.02 (-0.10,0.13) | 0.02 (-0.10,0.13) | -0.04 (-0.13,0.05) | -0.03 (-0.12,0.05) |
|  | $20,000 or greater |  | 0.06 (-0.05,0.16) | 0.06 (-0.05,0.16) | 0.06 (-0.05,0.16) | 0.06 (-0.05,0.16) | 0.00 (-0.09,0.09) | 0.01 (-0.08,0.10) |
| Any household member works for pay | |  | -0.01 (-0.12,0.10) | -0.01 (-0.12,0.10) | -0.01 (-0.12,0.10) | -0.01 (-0.12,0.10) | 0.00 (-0.09,0.10) | 0.00 (-0.09,0.09) |
| Married or living as married | |  | 0.05 (-0.06,0.15) | 0.05 (-0.06,0.15) | 0.05 (-0.06,0.15) | 0.05 (-0.06,0.15) | -0.02 (-0.10,0.06) | -0.02 (-0.10,0.05) |
| Number of children in the household (ref. = No children) | |  |  |  |  |  |  |  |
|  | One or two children in the household |  | 0.02 (-0.09,0.13) | 0.02 (-0.09,0.13) | 0.02 (-0.09,0.13) | 0.02 (-0.09,0.13) | -0.02 (-0.11,0.07) | -0.03 (-0.11,0.06) |
|  | Three or more children in the household |  | -0.03 (-0.17,0.10) | -0.03 (-0.17,0.10) | -0.03 (-0.17,0.11) | -0.03 (-0.17,0.11) | -0.01 (-0.13,0.10) | -0.02 (-0.13,0.09) |
| Foreign born | |  | 0.07 (-0.06,0.21) | 0.07 (-0.06,0.21) | 0.07 (-0.06,0.21) | 0.07 (-0.06,0.21) | 0.06 (-0.04,0.17) | 0.07 (-0.04,0.18) |
| Ever been infected with COVID-19 | |  |  |  | 0.00 (-0.09,0.08) | 0.00 (-0.09,0.08) | 0.04 (-0.04,0.11) | 0.04 (-0.04,0.11) |
| Ever been diagnosed with cardiometabolic condition | |  |  |  | 0.01 (-0.07,0.10) | 0.02 (-0.07,0.10) | -0.02 (-0.09,0.06) | -0.02 (-0.10,0.05) |
| Have obesity | |  |  |  | -0.01 (-0.09,0.07) | -0.01 (-0.09,0.07) | 0.02 (-0.05,0.08) | 0.02 (-0.05,0.08) |
| Difficulties with activities of daily living | |  |  |  | 0.00 (-0.10,0.09) | 0.00 (-0.10,0.10) | 0.00 (-0.08,0.08) | 0.00 (-0.08,0.07) |
| COVID-19 problem scale | |  |  |  |  | 0.00 (-0.02,0.01) | 0.00 (-0.01,0.01) | 0.00 (-0.01,0.01) |
| Trust the process to develop safe COVID-19 vaccine | |  |  |  |  |  | 0.29*** (0.21,0.38) | 0.29*** (0.20,0.37) |
| COVID-19 vaccine provides important benefits to society | |  |  |  |  |  | 0.26*** (0.17,0.34) | 0.25*** (0.17,0.34) |
| Disagree that COVID-19 vaccine leads to illness or death | |  |  |  |  |  | 0.11** (0.04,0.18) | 0.11** (0.04,0.18) |
| Health insurance | |  |  |  |  |  |  | 0.04 (-0.07,0.16) |
| Access to health care | |  |  |  |  |  |  | 0.12 (-0.01,0.24) |
| Constant | | 0.34*** (0.26,0.43) | 0.19 (-0.00,0.37) | 0.19 (-0.00,0.37) | 0.19 (-0.00,0.38) | 0.20* (0.00,0.40) | 0.01 (-0.16,0.18) | -0.11 (-0.31,0.08) |
| N | | 550 | 550 | 550 | 550 | 550 | 548 | 548 |
| R2 | | 0.13 | 0.222 | 0.222 | 0.223 | 0.223 | 0.457 | 0.464 |
|  |  |  |  |  |  |  |  |  |
| * p < 0.05, ** p < 0.01, *** p < 0.001 | |  |  |  |  |  |  |  |

| **Table S2. Regression Coefficients from Linear Probability Models Predicting Vaccination Status (Using Alternative Measures of COVID-19 Attitudes)** | | | | | | | | |
| --- | --- | --- | --- | --- | --- | --- | --- | --- |
|  |  | Model 0 | Model 1 | Model 2 | Model 3 | Model 4 | Model 5 | Model 6 |
| Race, Ethnicity, Ancestry (ref. = Non-Hispanic Black) | | 0.31*** (0.21,0.41) | 0.30*** (0.18,0.42) | 0.30*** (0.18,0.42) | 0.31*** (0.19,0.42) | 0.31*** (0.19,0.43) | 0.20*** (0.09,0.30) | 0.24*** (0.13,0.35) |
|  | Hispanic, Mexican ancestry | 0.44*** (0.32,0.56) | 0.35*** (0.20,0.49) | 0.35*** (0.20,0.49) | 0.35*** (0.20,0.49) | 0.35*** (0.20,0.50) | 0.21*** (0.09,0.33) | 0.23*** (0.10,0.36) |
|  | Hispanic, non-Mexican ancestry | 0.09 (-0.00,0.19) | 0.08 (-0.01,0.17) | 0.08 (-0.01,0.17) | 0.08 (-0.01,0.18) | 0.08 (-0.01,0.18) | 0.09* (0.01,0.17) | 0.10* (0.02,0.19) |
|  | Survey completed December 2021 or later |  |  |  |  |  |  |  |
| Age (ref. = 18-34) | |  |  |  |  |  |  |  |
|  | 35-54 |  | 0.10 (-0.01,0.20) | 0.10 (-0.01,0.20) | 0.10 (-0.01,0.20) | 0.10 (-0.01,0.20) | 0.02 (-0.06,0.11) | 0.03 (-0.07,0.12) |
|  | 55+ |  | 0.34*** (0.21,0.47) | 0.34*** (0.21,0.47) | 0.34*** (0.19,0.48) | 0.33*** (0.19,0.48) | 0.20*** (0.09,0.32) | 0.20** (0.07,0.32) |
| Female | |  | 0.01 (-0.08,0.10) | 0.01 (-0.08,0.10) | 0.01 (-0.08,0.10) | 0.01 (-0.08,0.10) | 0.05 (-0.03,0.12) | 0.02 (-0.06,0.10) |
| Education (ref. = Less than high school) | |  |  |  |  |  |  |  |
|  | High school |  | 0.00 (-0.10,0.09) | 0.00 (-0.10,0.10) | 0.00 (-0.10,0.09) | 0.00 (-0.10,0.09) | 0.02 (-0.05,0.10) | 0.01 (-0.07,0.09) |
|  | More than HS |  | 0.00 (-0.11,0.11) | 0.00 (-0.11,0.11) | -0.01 (-0.12,0.10) | 0.00 (-0.11,0.11) | 0.08 (-0.01,0.16) | 0.08 (-0.02,0.17) |
| Household Income (ref. = Less than $10,000) | |  |  |  |  |  |  |  |
|  | $10,000-$19,999 |  | 0.03 (-0.08,0.14) | 0.03 (-0.08,0.14) | 0.03 (-0.08,0.14) | 0.03 (-0.08,0.14) | -0.03 (-0.11,0.06) | -0.03 (-0.12,0.05) |
|  | $20,000 or greater |  | 0.06 (-0.05,0.17) | 0.06 (-0.05,0.17) | 0.06 (-0.05,0.17) | 0.06 (-0.05,0.17) | -0.01 (-0.09,0.08) | 0.01 (-0.08,0.10) |
| Any household member works for pay | |  | -0.01 (-0.12,0.09) | -0.01 (-0.12,0.09) | -0.02 (-0.13,0.09) | -0.02 (-0.13,0.09) | 0.00 (-0.09,0.09) | 0.00 (-0.09,0.09) |
| Married or living as married | |  | 0.06 (-0.04,0.16) | 0.06 (-0.04,0.16) | 0.06 (-0.04,0.16) | 0.06 (-0.04,0.16) | -0.01 (-0.08,0.07) | -0.02 (-0.10,0.05) |
| Number of children in the household (ref. = No children) | |  |  |  |  |  |  |  |
|  | One or two children in the household |  | 0.02 (-0.09,0.13) | 0.02 (-0.09,0.13) | 0.02 (-0.09,0.13) | 0.02 (-0.09,0.13) | -0.02 (-0.10,0.07) | -0.03 (-0.11,0.06) |
|  | Three or more children in the household |  | -0.02 (-0.16,0.11) | -0.02 (-0.16,0.11) | -0.03 (-0.17,0.11) | -0.03 (-0.17,0.11) | -0.02 (-0.12,0.09) | -0.02 (-0.13,0.09) |
| Foreign born | |  | 0.08 (-0.05,0.20) | 0.08 (-0.05,0.20) | 0.08 (-0.05,0.20) | 0.08 (-0.05,0.20) | 0.06 (-0.04,0.16) | 0.07 (-0.04,0.18) |
| Ever been infected with COVID-19 | |  |  |  | 0.00 (-0.09,0.08) | 0.00 (-0.09,0.08) | 0.03 (-0.04,0.10) | 0.04 (-0.04,0.11) |
| Ever been diagnosed with cardiometabolic condition | |  |  |  | 0.03 (-0.05,0.12) | 0.03 (-0.05,0.12) | -0.01 (-0.08,0.06) | -0.02 (-0.10,0.05) |
| Have obesity | |  |  |  | -0.02 (-0.10,0.06) | -0.02 (-0.09,0.06) | 0.01 (-0.05,0.08) | 0.02 (-0.05,0.08) |
| Difficulties with activities of daily living | |  |  |  | -0.04 (-0.14,0.06) | -0.04 (-0.14,0.06) | -0.02 (-0.09,0.05) | 0.00 (-0.08,0.07) |
| COVID-19 problem scale | |  |  |  |  | 0.00 (-0.02,0.01) | 0.00 (-0.01,0.01) | 0.00 (-0.01,0.01) |
| Trust the process to develop safe COVID-19 vaccine | |  |  |  |  |  | 0.30*** (0.22,0.38) | 0.29*** (0.20,0.37) |
| COVID-19 vaccine provides important benefits to society | |  |  |  |  |  | 0.28*** (0.20,0.36) | 0.25*** (0.17,0.34) |
| Disagree that COVID-19 vaccine leads to illness or death | |  |  |  |  |  | 0.10** (0.04,0.17) | 0.11** (0.04,0.18) |
| Health insurance | |  |  |  |  |  |  | 0.04 (-0.07,0.16) |
| Access to health care | |  |  |  |  |  |  | 0.12 (-0.01,0.24) |
| Constant | | 0.35*** (0.27,0.43) | 0.18* (0.01,0.35) | 0.18* (0.01,0.36) | 0.19* (0.01,0.37) | 0.21* (0.02,0.39) | 0.00 (-0.15,0.16) | -0.11 (-0.31,0.08) |
| N | | 616 | 616 | 616 | 616 | 616 | 614 | 548 |
| R2 | | 0.112 | 0.209 | 0.209 | 0.211 | 0.211 | 0.466 | 0.464 |
|  |  |  |  |  |  |  |  |  |
| * p < 0.05, ** p < 0.01, *** p < 0.001 | |  |  |  |  |  |  |  |

**Figure S1. Survey Participation Dates by Race/Ethnicity**


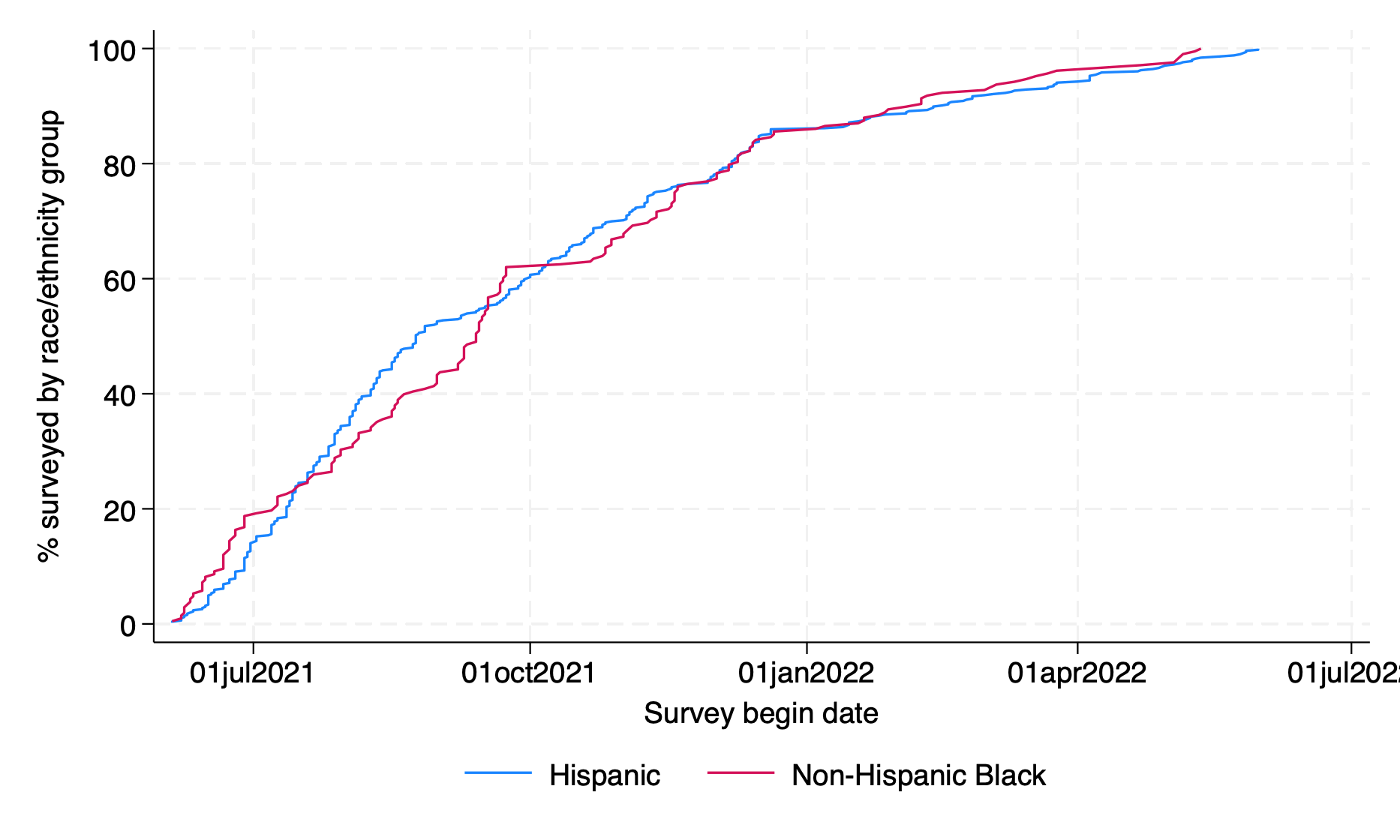


| **Table S3. Predicted Proportion Vaccinated for COVID-19 across Racial and Ethnic Groups** | | | | | | | | |
| --- | --- | --- | --- | --- | --- | --- | --- | --- |
|  |  | Model 0 | Model 1 | Model 2 | Model 3 | Model 4 | Model 5 | Model 6 |
| Panel A. Linear Probability | |  |  |  |  |  |  |  |
|  | Non-Hispanic Black | 0.37 (0.29,0.44) | 0.38 (0.30,0.47) | 0.38 (0.29,0.47) | 0.38 (0.29,0.47) | 0.38 (0.29,0.47) | 0.47 (0.39,0.55) | 0.47 (0.38,0.55) |
|  | Hispanic, Mexican ancestry | 0.67 (0.61,0.74) | 0.69 (0.62,0.75) | 0.69 (0.62,0.75) | 0.69 (0.62,0.75) | 0.69 (0.63,0.75) | 0.66 (0.61,0.71) | 0.69 (0.64,0.74) |
|  | Hispanic, non-Mexican ancestry | 0.81 (0.72,0.90) | 0.73 (0.63,0.83) | 0.73 (0.63,0.83) | 0.73 (0.63,0.83) | 0.73 (0.63,0.83) | 0.69 (0.60,0.77) | 0.69 (0.60,0.78) |
|  |  |  |  |  |  |  |  |  |
| Panel B. Logistic Regression | |  |  |  |  |  |  |  |
|  | Non-Hispanic Black | 0.37 (0.29,0.44) | 0.39 (0.30,0.48) | 0.39 (0.30,0.48) | 0.39 (0.30,0.48) | 0.39 (0.30,0.48) | 0.47 (0.39,0.55) | 0.48 (0.39,0.56) |
|  | Hispanic, Mexican ancestry | 0.67 (0.61, 0.74) | 0.69 (0.63,0.74) | 0.69 (0.63,0.75) | 0.69 (0.63,0.74) | 0.69 (0.63,0.74) | 0.66 (0.61,0.70) | 0.69 (0.64,0.74) |
|  | Hispanic, non-Mexican ancestry | 0.81 (0.72, 0.90) | 0.75 (0.64,0.86) | 0.75 (0.64,0.86) | 0.75 (0.64,0.86) | 0.75 (0.64,0.86) | 0.69 (0.61,0.77) | 0.70 (0.62,0.78) |
